# Sorafenib resistance in hepatocarcinoma: role of hypoxia-inducible factors

**DOI:** 10.1038/s12276-018-0159-1

**Published:** 2018-10-12

**Authors:** Carolina Méndez-Blanco, Flavia Fondevila, Andrés García-Palomo, Javier González-Gallego, José L. Mauriz

**Affiliations:** 1 Institute of Biomedicine, University of León, León, Spain; 2 Centro de Investigación Biomédica en Red de Enfermedades Hepáticas y Digestivas (CIBERehd), Madrid, Spain; 3 Service of Oncology, Complejo Asistencial Universitario de León, León, Spain

## Abstract

Sorafenib, a multikinase inhibitor with antiproliferative, antiangiogenic, and proapoptotic properties, constitutes the only effective first-line drug approved for the treatment of advanced hepatocellular carcinoma (HCC). Despite its capacity to increase survival in HCC patients, its success is quite low in the long term owing to the development of resistant cells through several mechanisms. Among these mechanisms, the antiangiogenic effects of sustained sorafenib treatment induce a reduction of microvessel density, promoting intratumoral hypoxia and hypoxia-inducible factors (HIFs)-mediated cellular responses that favor the selection of resistant cells adapted to the hypoxic microenvironment. Clinical data have demonstrated that overexpressed HIF-1α and HIF-2α in HCC patients are reliable markers of a poor prognosis. Thus, the combination of current sorafenib treatment with gene therapy or inhibitors against HIFs have been documented as promising approaches to overcome sorafenib resistance both in vitro and in vivo. Because the depletion of one HIF-α subunit elevates the expression of the other HIF-α isoform through a compensatory loop, targeting both HIF-1α and HIF-2α would be a more interesting strategy than therapies that discriminate among HIF-α isoforms. In conclusion, there is a marked correlation between the hypoxic microenvironment and sorafenib resistance, suggesting that targeting HIFs is a promising way to increase the efficiency of treatment.

## Liver cancer: overcoming treatment resistance

Targeting hypoxia-inducible factors (HIFs), regulatory proteins induced by low oxygen levels, could increase the effectiveness of sorafenib, the only systemic therapy approved for advanced liver cancer. Long-term treatment with sorafenib starves tumors of oxygen, which can lead to the proliferation of cancer cells that are able to survive low oxygen levels. HIFs regulate genes involved in this adaptation and HIF levels are increased in sorafenib-resistant cells. José Mauriz at the University of León, Spain, and colleagues review recent studies on the effects of HIF inhibition on sorafenib efficacy. They conclude that HIF-1α and HIF-2α are predictive markers of sorafenib resistance and that using inhibitors of both these factors as an add-on therapy could improve patient survival. This strategy may be applicable to other types of cancer in which reduced oxygen conditions lead to drug resistance.

## Introduction

Hepatocellular carcinoma (HCC) represents the second and the sixth cause of cancer-related death worldwide in men and women, respectively, and its incidence is increasing in regions with historically low rates such as Oceania, Western Europe and Northern America^1^. Unfortunately, most cases are diagnosed in advanced stages, when there are no amenable curative therapies, and the unique palliative drug approved by the Food and Drug Administration (FDA) as the first-line chemotherapy is sorafenib (BAY 43–9006, Nexavar^®^; Bayer HealthCare Pharmaceuticals Inc.; Leverkusen, Germany)^2,3^.

Sorafenib is a multikinase inhibitor that blocks tumor cell proliferation by inhibiting serine/threonine kinase isoforms of Raf, Raf-1, and B-Raf, leading to the inhibition of mitogen-activated protein kinase/extracellular signal-regulated kinase (ERK) signaling pathways, decreased expression of cyclin D1 and cell cycle arrest^4–6^. Sorafenib exhibits antiangiogenic activity by targeting the tyrosine kinase receptors hepatocyte factor receptor (c-Kit), FMS-like tyrosine kinase (FLT-3), vascular endothelial growth factor receptors 2 and 3 (VEGFR-2, VEGFR-3), and platelet-derived growth factor receptor^4,7,8^. In addition, sorafenib promotes apoptosis by inhibiting eIF4E phosphorylation and subsequent downregulation of the antiapoptotic factor Mcl-1 translation^4,7^ or through a progressive increase in endoplasmic reticulum stress associated with a shift from autophagy to apoptosis^9^.

Sorafenib approval was founded on the results of the Sorafenib Hepatocellular Carcinoma Assessment Randomized Protocol (SHARP) trial, in which it was shown to be effective and safe. SHARP was an international, multicenter, randomized, double-blind, placebo-controlled trial in 602 patients with unresectable HCC. This trial demonstrated that sorafenib could improve patient survival; however, the response rates were very low (from 7.9 months in the placebo group to 10.7 with sorafenib; hazard ratio: 0.69 (95% CI: 0.55, 0.87), *p* = 0.00058). In addition to its clinical benefits, sorafenib is usually well tolerated, with fatigue, weight loss, rash/desquamation, alopecia, palmar-plantar erythrodysesthesia, diarrhea, anorexia, nausea, and abdominal pain being the more common adverse reactions^10^. These results were supported by a phase III randomized, double-blind, placebo-controlled trial in 271 patients of the Asia-Pacific region^11^ and by the Global Investigation of therapeutic DEcisions in HCC and Of its treatment with sorafeNib (GIDEON), which included a heterogeneous population of 1571 unresectable HCC patients^12^. In recent years, various clinical studies have also indicated that sorafenib has antitumor effects not only in HCC but also in other cancer types, including thyroid cancer, myeloid leukemia, mesothelioma, renal cell carcinoma, and prostate cancer^13–17^.

Although sorafenib can prolong survival in HCC patients, its efficacy is short owing to the development of resistant cells. Although some patients are initially resistant to sorafenib because of HCC heterogeneity, in most cases, the resistance is acquired because of long-term exposure to the drug. Several mechanisms are implicated in the reduction of tumor cell sensitivity to sorafenib, such as loops of the phosphatidylinositol-3-kinase (PI3K)/protein kinase B (Akt) and janus tyrosine kinase (JAK)/signal transducer and activator of transcription (STAT) pathways, epithelial–mesenchymal transition (EMT) or hypoxia-inducible response^5,8,18,19^. Here, we consider the role of the hypoxic microenvironment in sorafenib resistance.

## Hypoxia and HIFs in HCC

The tumor microenvironment is closely involved in tumor development^8^. Changes in the oxygen supply that occur during inflammation, metabolic disorders, steatohepatitis, viral hepatitis, and carcinogenesis are sufficient to promote a hypoxic response. However, despite the variable oxygen tensions, hypoxic responses are not observed in normal healthy liver^20^. Hypoxia is a common property of solid tumors, such as HCC, which appears because of faulty vascularization and intense metabolic activity, related to radio- and chemoresistance, selection of more invasive clones and poor clinical outcomes^8,20,21^. The adaptive response to hypoxia entails a set of “prosurvival” changes regulated by hypoxia-inducible factors (HIFs) and is involved in tumor development and progression^22,23^.

HIFs are transcription factors that regulate a wide range of genes involved in proliferation, glucose metabolism, angiogenesis, tumor invasion, and metastasis; all processes focused on cell adaptation to the lack of oxygen^20,23^. HIFs are heterodimeric complexes comprising a HIF-α subunit regulated through oxygen-dependent proteasomal degradation and a HIF-β subunit constitutively expressed^24^. Three isoforms of the HIF-α subunit (HIF-1α, HIF-2α, and HIF-3α) have been described, and the overexpression of HIF-1α and HIF-2α has been detected in different liver diseases, including nonalcoholic fatty liver disease, alcoholic liver disease, radiation-induced liver injury, and HCC^23^.

HIF-1α and HIF-2α share a similar protein structure and mutual targets, but they regulate independent patterns of downstream gene induction; although HIF-1α is ubiquitously expressed, HIF-2α is only expressed by definite cell types, including hepatocytes^25,26^. The cellular levels of HIF-α subunits depend on the balance between its oxygen-dependent degradation and oxygen-independent synthesis^21,24^. Under normoxia (normal oxygen supply), HIF-α is constitutively degraded and kept at low basal activity^24^. Prolyl hydroxylases (PHDs) hydroxylate proline residues using oxygen as cofactor and allow the interaction between HIF-α and the von Hippel-Lindau (VHL) tumor suppressor protein. Successively, the ubiquitin E3 ligase protein recognizes VHL, resulting in the ubiquitination of HIF-α and its imminent proteasomal degradation^21,23^. Furthermore, factor inhibiting HIF hydroxylates asparagine residues of HIF-α, disturbing the interaction between HIF and the transcriptional coactivators CREB-binding protein and p300 in the promoter regions^24^. Conversely, under hypoxia conditions (low oxygen supply), hydroxylation, and proteasomal degradation of HIF-α weaken owing to the lack of oxygen, so that HIF-α is stabilized and translocated into the nucleus, where it heterodimerizes with HIF-β and binds to hypoxia-response elements in the promoters of its targets genes involved in tumor progression and therapy resistance^20,24^.

During the long-term periods of hypoxia, a HIF-1α-dependent feedback loop increases PHDs expression, leading to the reactivation of HIF-1α hydroxylation and proteasomal degradation. Hence, HIF-1α appears to play the main role in the response to acute hypoxia, whereas the HIF-2α levels may increase over time, driving the response to chronic hypoxia^20,26^. In addition, the depletion of one HIF-α subunit elevates the levels of the other HIF-α isoforms by a compensatory loop, and it is known that the switch from HIF-1α to HIF-2α confers to the tumor a more aggressive phenotype^26^.

It was demonstrated that both HIF-1α and HIF-2α are upregulated in HCC and are considered markers of poor prognosis. However, studies have also reported that overexpression of HIF-2α could play a tumor suppressor role in HCC depending on the cellular context^27^.

## HIFs and sorafenib resistance

Lack of oxygen is common in solid tumors such as HCC and drives vascular endothelial growth factor (VEGF) production and angiogenesis through HIF-1α activation^28^. Thus, the antiangiogenic actions of sorafenib are derived from blockade of the HIF-1α/VEGF pathway^29–31^. Sorafenib inhibits hypoxia-induced HIF-1α protein synthesis, leading to decreased VEGF expression and lower tumor vascularization both in different HCC cell lines^29,31^ and HCC xenograft mice^29^. A study by Xu et al.^30^ reported that this drug could reduce HIF-1α and VEGF expression and microvessel density, augmenting the time to recurrence when it is used as a coadjuvant to radiofrequency ablation.

**Fig. 1:**
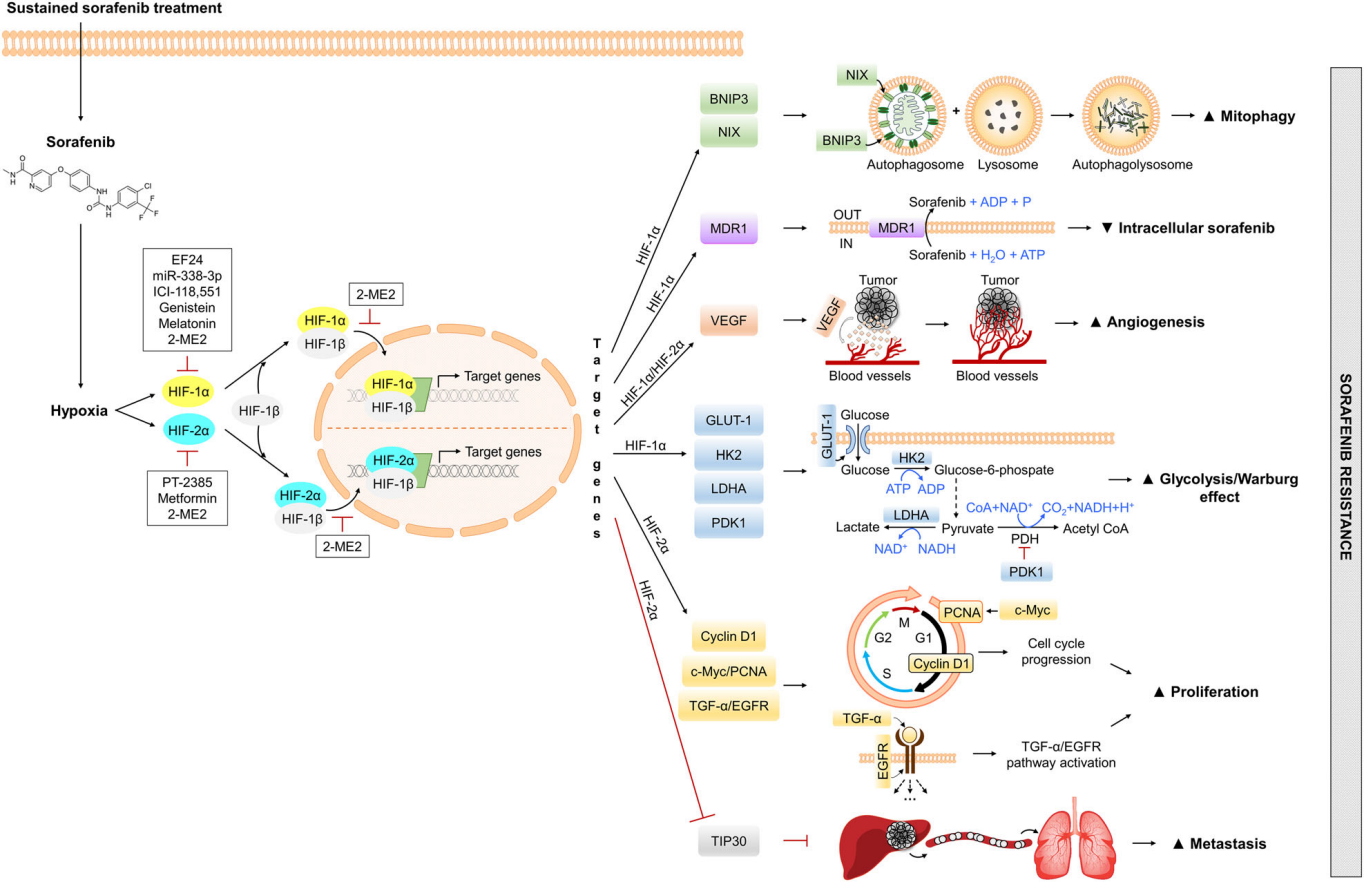
Hypoxia-related mechanisms of sorafenib resistance and targeting strategies against HIFs Sustained sorafenib treatment enhances hypoxia-inducible factors 1α or 2α, which promote the transcription of a wide range of genes involved in mitophagy, proliferation, glucose metabolism, angiogenesis, tumor invasion, and metastasis, leading to sorafenib resistance. This resistance can be overcome by different small molecules or drugs that inhibit HIFs. ADP, adenosine diphosphate; ATP, adenosine triphosphate; BNIP3, B-cell lymphoma-2 (BCL2)/adenovirus E1B 19 kDa-interacting protein 3; c-Myc, Myc proto-oncogene protein; CoA, coenzyme A; EGFR, phospho-epidermal growth factor receptor; GLUT-1, glucose transporter 1; HIF, hypoxia-inducible factor; HK2, hexokinase 2; LDHA, lactate dehydrogenase A; MDR1, multidrug resistance protein 1; NAD^+^, nicotinamide adenine dinucleotide (oxidized form); NADH, nicotinamide adenine dinucleotide (reduced form); NIX, BNIP3-like protein X; P, phosphate; PCNA, proliferating cell nuclear antigen; PDH, pyruvate dehydrogenase; PDK1, pyruvate dehydrogenase kinase isoform 1; TGF-α, transforming growth factor α; TIP30, oxidoreductase HTATIP2; VEGF, vascular endothelial growth factor

Nonetheless, there is a gripping correlation between acquired sorafenib resistance and the hypoxic microenvironment because the antiangiogenic activity of sustained sorafenib treatment leads to tumor starvation and succeeding intratumoral hypoxia, favoring the selection of resistant cell clones adapted to the deficit of oxygen and nutrients^32–34^. This situation limits sorafenib efficiency (Fig. 1)^32^.

It was reported that hypoxia confers sorafenib resistance in myeloid leukemia, renal, or gastric cancer cells^35–38^ and is responsible for the acquired resistance to different anticancer drugs in HCC cells, including doxorubicin, etoposide, cisplatin, SN38, and 5-fluorouracil^39–43^. In fact, Liang et al.^32^ reported that the continued administration of sorafenib in HCC subcutaneous mouse tumor models increases the protein levels and transcriptional activity of HIF-1α. Likewise, HCC tissues obtained from sorafenib-resistant patients display increased intratumor hypoxia and expression of HIF-1α compared with sorafenib-sensitive or untreated HCCs^32^. Sorafenib resistance is associated with the increased expression of the multidrug resistance protein 1 (MDR1), glucose transporter 1 (GLUT-1), and VEGF because of HIF-1α protein stabilization^32,44^. In addition, galectin-1, a protein involved in modulating cell–cell and cell–matrix interactions, was reported to be a downstream target of the Akt/mTOR/HIF-1α pathway and was defined as a predictive marker of sorafenib resistance^45^. β-2 Adrenergic receptor (ADRB2) signaling modulates autophagy negatively by disturbing the beclin1/phosphatidylinositol-3-kinase VPS3/autophagy-related protein 14 complex in an Akt-dependent manner, promoting the stabilization of HIF-1α, reprogramming glucose metabolism of HCC cells, and leading to the acquisition of sorafenib resistance^46^. This role of glycolysis in sorafenib resistance was supported by a report where HIF-1α activation endorses cell survival by amplifying the expression of glycolytic enzymes—for instance, GLUT-1 and hexokinase 2 (HK2)—to accelerate the glycolytic rate^47^. Mitophagy, a specific form of autophagy, is activated under hypoxic conditions in HCC cells by the upregulation of the mitophagy targets of HIF-1α, B-cell lymphoma-2/adenovirus E1B 19 kDa-interacting protein 3 (BNIP3) and BNIP3-like protein X (NIX). It was described that mitophagy exerts a cytoprotective role on tumor cells; however, unfortunately, sorafenib treatment cannot abolish this process^33^.

In addition, given the feedback mechanism between the HIF-1α and HIF-2α subunits, it can be thought that sorafenib treatment may upregulate HIF-2α through the inhibition of HIF-1α, promoting sorafenib resistance and a more aggressive tumor growth^25,26,34^. It was verified that sorafenib upregulates HIF-2α through the hypoxic response switch from HIF-1α inhibition, contributing to the resistance of hypoxic HCC cells by activating the HIF-2α/transforming growth factor (TGF)-α/epidermal growth factor receptor (EGFR) pathway^26^ and increasing the expression of VEGF and cyclin D1^25^. Another study found that sorafenib treatment enhances HIF-2α accumulation, contributing to androgen receptor (AR) reduction, which is related to HCC progression and metastasis^48^. Moreover, Liu et al.^34^ reported a feedback mechanism and described that the HIF-2α increase positively regulates β-catenin/Myc proto-oncogene protein (c-Myc) expression and that c-Myc directly upregulates proliferating cell nuclear antigen (PCNA) expression under hypoxic conditions in HCC cells, thus increasing the proliferation involved in sorafenib resistance. Zhu et al.^49^ also supported that HIF-2α is involved in the process by which hypoxia protects HCC cells against sorafenib. Hepatopoietin Cn (HPPCn) is a growth factor isolated from hepatic stimulator substance that promotes sorafenib resistance by elevating HIF-2α levels through the promotion of cell growth and metastasis in HCC. In addition, Akt-mediated sentrin-specific protease 1 (SENP1) upregulation accounts for HPPCn-induced HIF-2α accumulation under a hypoxic microenvironment. Another study confirmed that the overexpression of HIF-2α by sorafenib promotes HCC invasion and metastasis via the downregulation of oxidoreductase HTATIP2 (TIP30)^50^.

These studies endorse the existing relationship between the high expression of HIFs and the resistance phenomenon to sorafenib, suggesting that hypoxia significantly affects sorafenib therapy and that a promising way to overcome resistance is to target these factors (Table 1).

**Table 1:** Hypoxia and sorafenib resistance in HCC

Cell lines/animal models/human samples	Effects on HIF after sorafenib treatment	Global effects	References
HCCs from patients HepG2, Huh7, PLC-5, Hep3B, and SK-Hep-1 cells BALB/c mice inoculated with Hep3B or Huh7 and orthotopic Huh7 hepatic tumors	↑ HIF-1α	↑ GLUT-1, MDR1, and VEGF Activation of NF-κB	32
Human HCC samples HepG2, SMMC-7721, BEK-7402, Hep3B, and Huh7 cells BALB/c nude mice subcutaneous model with HepG2 cells	↑ HIF-1α	↑ GLUT-1, MDR1, and VEGF	44
Sorafenib-resistant Huh7 cells (Huh7^R^) Tumor xenograft model by subcutaneously injecting Huh7 and Huh7^R^ cells in BALB/c nude mice	↑ HIF-1α	↑ Galectin-1	45
HCC samples HepG2, SMMC-7721, MHCC-LM3, and CSQT-2 cells DEN-induced HCC in C57BL/6 mice Mouse xenograft model derived from SMMC-7721 cells	↑ HIF-1α	↑ GLUT-1, HK2, PDK1, LDHA, and VEGF	46
HCC-LM3 and Bel-7402 cells BALB/c nu/nu injected with HCC-LM3 cells	↑ HIF-1α	↑ GLUT-1 and HK2	47
HepG2 and Huh7 cells BALB/c mice with Huh7 subcutaneously injected	↓ HIF-1α ↑ HIF-2α	Activation of TGF-α/EGFR	26
HepG2 and Huh7 cells BALB/c mice with Huh7 subcutaneously injected	↓ HIF-1α ↑ HIF-2α	↑ VEGF and cyclin D1 ↓ LDHA	25
HepG2, Huh7, and SK-Hep-1 cells Mice HCC nodules Orthotopic HCC mouse models with SK-Hep-1 cells	↑ HIF-2α	↑ AR	25
HepG2, Huh7, Bel-7402, and SMMC-7402 cells BALB/c mice subcutaneously inoculated with HepG2	↓ HIF-1α ↑ HIF-2α	↑ β-catenin/c-Myc/PCNA	34
HCC cells	↑ HIF-2α	–	49
MHCC97H cells BALB/c mice subcutaneously injected with MHCC97H cells	↑ HIF-2α	↓ TIP30	50

*AR* androgen receptor, *c-Myc* Myc proto-oncogene protein, *DEN* diethylnitrosamine, *EGFR* epidermal growth factor receptor, *GLUT-1* glucose transporter 1, *HCC* hepatocellular carcinoma, *HIF* hypoxia-inducible factor, *HK2* hexokinase 2, *LDHA* lactate dehydrogenase A, *MDR1* multidrug resistance protein 1, *PCNA* proliferating cell nuclear antigen, *PDK1* pyruvate dehydrogenase kinase isoform 1, *TGF-α* transforming growth factor α, *TIP30* oxidoreductase HTATIP2, *VEGF* vascular endothelial growth factor

## Targeting HIFs to overcome sorafenib resistance

Given the pathological role of HIFs in liver disease, particularly in HCC, inhibition of HIFs is being explored as an effective treatment strategy. Furthermore, because of the participation of HIFs in the development of resistance to chemotherapeutic drugs in HCC, HIF inhibitors can be administered in combination with current therapies (Table 2)^23^.

**Table 2:** Therapeutic approaches against HIFs to overcome sorafenib resistance in HCC

Models	Strategies to target hypoxia markers	Effects on hypoxia markers	Global effects	References
HepG2, Huh7, PLC-5, Hep3B, and SK-Hep-1 cells BALB/c mice inoculated with Hep3B or Huh7 and orthotopic Huh7 hepatic tumors	EF24 plus sorafenib	VHL-dependent HIF-1α protein degradation	↓ DNA-binding activity of NF-κB ↓ VEGF, GLUT-1 ↓ Migration, invasion, and metastasis ↑ Apoptosis	32
HepG2, SMMC-7721, BEK-7402, Hep3B, and Huh7 cells BALB/c nude mice subcutaneous model with HepG2 cells	Overexpression of miR-338-3p plus sorafenib treatment	HIF-1α mRNA inhibition	↓ MDR1 ↓ Tumor growth ↑ Apoptosis	44
HepG2, SMMC-7721, MHCC-LM3, and CSQT-2 cells Mouse xenograft model derived from SMMC-7721 cells	ICI-118,551 plus sorafenib	Autophagic degradation of HIF-1α protein	↓ Glucose metabolism and proliferation ↑ Autophagic cell death	46
HCC-LM3 and Bel-7402 cells BALB/c nu/nu injected with HCC-LM3 cells	Genistein plus sorafenib	HIF-1α mRNA downregulation	↓ GLUT-1 and HK2 ↓ Glycolysis and induce apoptosis	47
Hep3B cells	Melatonin and sorafenib combination	HIF-1α protein downregulation	↓ BNIP3 and NIX ↓ Cytoprotective hypoxia-induced mitophagy	33
HepG2 and Huh7 cells BALB/c mice with Huh7 subcutaneously injected	HIF-2α siRNA plus sorafenib	Knockdown of HIF-2α by RNA interference	↓ HIF-2α, TGF-α, p-EGFR, p-STAT3, p-Akt, p-ERK, cyclin D1, and VEGF ↓ HCC tumors growth ↑ Apoptosis	26
HepG2, Huh7, and SK-Hep-1 cells Orthotopic HCC mouse models with SK-Hep-1 cells	PT-2385 plus sorafenib	HIF-2α protein downregulation	↑AR ↓ p-STAT3, p-Akt, p-ERK, B-Raf, and Raf-1 ↓ Invasion	48
HCC cells	HIF-2α shRNA plus sorafenib	Knockdown of HIF-2α by RNAinterference	↓ Tumor growth and metastasis	49
HepG2, Huh7, Bel-7402, and SMMC-7402 cells BALB/c mice subcutaneously inoculated with HepG2	HIF-2α shRNA or siRNA plus sorafenib	Knockdown of HIF-2α by RNA interference	↓ β-catenin/c-Myc/PCNA ↓ Proliferation and tumor growth	34
MHCC97H cells BALB/c mice subcutaneously injected with MHCC97H cells	Metformin plus sorafenib	HIF-2α protein inhibition	↑ TIP30 ↓ Proliferation, EMT, postoperative recurrence, and lung metastasis ↑ Apoptosis	50
HepG2 and Huh7 cells BALB/c mice with Huh7 subcutaneously injected	2-ME2 plus sorafenib	Inhibition of nuclear translocation and expression of HIF-1α and HIF-2α proteins	↓ VEGF, LDHA, and cyclin D1 ↓ Proliferation and angiogenesis ↑ Apoptosis	25

*2-ME2* 2-methoxyestradiol, *ADRB2 β-2* adrenergic receptor, *AR* androgen receptor, *B-Raf* serine/threonine-protein kinase, *BNIP3* B-cell lymphoma-2 (BCL2)/adenovirus E1B 19 kDa-interacting protein 3, *c-Myc* Myc proto-oncogene protein, *EMT* epithelial–mesenchymal transition, *GLUT-1* glucose transporter 1, *HCC* hepatocellular carcinoma, *HIF* hypoxia-inducible factor, *HK2* hexokinase 2, *LDHA* lactate dehydrogenase A, *MDR1* multidrug resistance protein 1, *NIX* BNIP3-like protein X, *p-Akt* phospho-protein kinase B, *p-EGFR* phospho-epidermal growth factor receptor, *p-ERK* phospho-extracellular signal-regulated kinases, p-STAT3 phospho-signal transducer and activator of transcription 3, *PCNA* proliferating cell nuclear antigen, *Raf-1* Raf proto-oncogene serine/threonine-protein kinase, *TGF-α* transforming growth factor α, *TIP30* oxidoreductase HTATIP2, *VEGF* vascular endothelial growth factor, *VHL* von Hippel-Lindau

Different studies showed that sustained sorafenib therapy leads to increased intratumor hypoxia, which has been associated with reduced sorafenib sensitivity through HIF stabilization in HCC, and reported that targeting HIF-1α can improve sorafenib efficacy. Liang et al.^32^ suggested that hypoxia induced by continued sorafenib treatment causes sorafenib resistance in HCC through HIF-1α and nuclear factor kappa B (NF-κB) activation. The combination of sorafenib and EF24, a curcumin analog, can overcome hypoxia-mediated sorafenib resistance by encouraging the proteasomal degradation of HIF-1α in a VHL-dependent manner in HCC cells, leading to the suppression of its target genes MDR1, GLUT-1, and VEGF and activity of NF-κB. The combination of EF24 and sorafenib also exhibited synergistic properties against tumor growth in subcutaneous and orthotopic hepatic tumor models^32^. In addition, the overexpression of miR-338-3p, which is strikingly downregulated in HCC patient samples and HCC cell lines, reduces cell viability and stimulates cell apoptosis by directly binding to the 3´-UTR of HIF-1α. Moreover, transfection of miR-338-3p can surpass sorafenib resistance mediated by hypoxia, acting synergistically against HCC tumor growth in an HCC subcutaneous nude mice tumor model by inhibiting HIF-1α^44^. ADRB2 signaling plays an essential role in maintaining the proliferation and survival of HCC cells through the stabilization of HIF-1α mediated by the downregulation of the autophagy process, leading to the reprogramming of glucose metabolism of HCC cells and acquisition of sorafenib resistance. Thus, the inhibition of ADRB2 signaling by the adrenoreceptor antagonist ICI-118,551 or knockdown of ADRB2 expression improved autophagy, which induced HIF-1α destabilization and upgraded the antitumor activity of sorafenib^46^.

The use of natural compounds has also shown positive effects in the improvement of sorafenib treatment. Genistein, a natural isoflavone, enhanced the antitumor effects of sorafenib in sorafenib-resistant HCC cells and an HCC xenograft mouse model by downregulating HIF-1α, therefore inactivating GLUT-1 and HK2 to suppress glycolysis and sensitize aerobic glycolytic HCC cells to the mitochondrial apoptosis^47^. Similarly, melatonin, a natural hormone, can downregulate HIF-1α protein synthesis through the inhibition of the mammalian target of rapamycin complex 1/ribosomal protein S6 kinase beta-1/ribosomal protein S6 pathway. Moreover, sorafenib and melatonin coadministration reduced the expression of HIF-1α-mitophagy targets BNIP3 and NIX, blocking the cytoprotective mitophagy induced by the hypoxic microenvironment^33^.

Nonetheless, other studies have focused their attention on the hypoxic response switch from HIF-1α- to HIF-2α-dependent pathways supported by sorafenib and succeeding the upregulation of HIF-2α, which subscribes to the insensitivity of hypoxic HCC cells to the drug^25^. Zhao et al.^26^ proposed that sorafenib-induced HIF-2α upregulation contributes to the resistance of hypoxic HCC cells by activating the TGF-α/EGFR pathway. The employment of gefitinib, a specific EGFR inhibitor, allows blockade of the TGF-α/EGFR pathway, downregulating the activation of STAT3, Akt, and ERK, and, in combination with sorafenib, can reduce the proliferation and induce the apoptosis of HCC cells under hypoxia. Likewise, transfection of HIF-2α siRNA decreased the expression of TGF-α, VEGF, and cyclin D1, and repressed the activation of EGFR, inhibiting the proliferation and promoting the apoptosis of HCC cells in vitro; HIF-2α siRNA also synergized with sorafenib to suppress the growth of HCC tumors in vivo^26^. Furthermore, sorafenib-induced or hypoxia-induced HIF-2α transcriptionally suppresses AR by binding to an HRE of the AR promoter. In vitro and in vivo studies have suggested that PT-2385, a specific HIF-2α inhibitor, improves sorafenib effectiveness by inhibiting HIF-2α, increasing AR and suppressing the downstream activation of STAT3, Akt, and ERK pathways^48^.

Another study also supported that HIF-2α contributes to sorafenib resistance in HCC. On this occasion, desumoylation of HIF-2α by SENP1 was reported to be implicated in HPPCn-enhanced sorafenib resistance under hypoxic conditions in HCC. The growth factor HPPCn increases HIF-2α levels, which promote cell growth and metastasis; thus, the combination of lentivirus-mediated HIF-2α shRNA and sorafenib presented synergistically effects to prevent tumor growth^49^. A study by Liu et al.^34^ showed that HIF-2α is involved in sorafenib resistance by regulating cell proliferation via the β-catenin/c-Myc-dependent pathway under hypoxic conditions. Thus, the combination of HIF-2α shRNA and sorafenib treatment exhibited an additive positive effect on inhibiting proliferation in HCC cells and in HepG2 xenograft mouse tumors, a finding mainly attributed to the decreased expression of PCNA that is directly regulated by c-Myc^34^.

It was also found that the overexpression of HIF-2α by sorafenib decreased the expression of TIP30, an oxidoreductase required for tumor suppression, stimulating the process of EMT and the subsequent promotion of HCC invasion and metastasis. Metformin (the first-line medication for the treatment of type II diabetes) has been tested in addition to sorafenib, showing inhibited expression of HIF-2α but upregulation of TIP30 at the protein levels, recovering the sensitivity of hypoxic HCC cells to sorafenib therapy in vitro. In addition, the combination of sorafenib with metformin revealed meaningful inhibition of the recurrence and metastasis of primary liver cancer in an orthotopic xenograft mouse model following surgical resection by modulating the expression of HIF-2α and TIP30^50^.

Unlike therapies that differentiate among HIF-α isoforms, targeting both HIF-1α and HIF-2α signaling would be more prejudicial to tumor cell survival than strategies directed only against one of them^20^. A study by Ma et al.^25^ employed 2-methoxyestradiol (2-ME2), a natural metabolite of estradiol. 2-ME2 repressed the nuclear translocation of HIF-1α and HIF-2α proteins and significantly weakened the expression levels of both HIF-1α and HIF-2α as well as their downstream targets VEGF, lactate dehydrogenase A, and cyclin D1, enhancing the sorafenib sensitivity of hypoxic HCC cells. Furthermore, 2-ME2 synergized with sorafenib to inhibit the proliferation and induction of apoptosis of HCC cells in vitro and in vivo and to inhibit tumor angiogenesis^25^.

## Conclusions and future perspectives

Sorafenib, a multitarget tyrosine kinase inhibitor, is the only effective first-line drug for the treatment of advanced HCC patients. Sorafenib resistance in HCC is a fact; nonetheless, the mechanisms that explain this resistance are complex and remain unclear. The genetic heterogeneity of HCC could explain the appearance of primary resistance. Thus, the identification of predictive biomarkers related to primary resistance to sorafenib will be very rewarding. Sorafenib targets several kinase pathways; thus, it can also simultaneously or consecutively activate additional switches and compensatory pathways—for instance, PI3K/Akt and JAK/STAT pathways, EMT, and tumor hypoxia—leading to acquired resistance.

Recent evidence has supported that hypoxia plays a key role in HCC development and therapy. In fact, clinical data have proven that hypoxia markers HIF-1α and HIF-2α are trustworthy indicators of the poor prognosis of HCC patients; even so, the role of HIF-2α depends on the cellular context.

The antiangiogenic effects of long-term sorafenib treatment promote decreased microvessel density and enhanced tumor hypoxia, which lead to HIF-mediated cellular responses triggering adaptive mechanisms to the hypoxic microenvironment. Not surprisingly, HIFs have been recognized as potential targets for HCC therapy. The use of gene therapy to target HIFs or the addition of HIF inhibitors to current therapies has improved their effectiveness. Particularly, in cancers such as HCC, in which there is a recognized overexpression of HIFs, existing drugs such as sorafenib or other antiangiogenic and vascular targeting molecules promote the activity of HIFs. Additional studies are necessary to select optimal therapeutic agents targeted against HIFs for enhanced clinical outcomes, considering the response switch between HIF-1α and HIF-2α.

Different clinical trials have examined the effect of targeting hypoxia in HCC. A recently completed phase I study evaluated the intravenous infusion effect of the HIF-1α mRNA antagonist RO7070179 in HCC patients failing to respond to systemic therapy (clinicaltrials.gov Identifier: NCT02564614). Other trials have focused on the use of hypoxia-activated prodrugs (HAPs), molecules that specifically target the hypoxic fractions of tumors. Thus, two early phase I/II trials analyzed different HAPs, TH-302 or PR104, in combination with sorafenib, in patients with advanced HCC that cannot be removed by surgery (clinicaltrials.gov Identifier: NCT01497444 and NCT00862082). A phase I study to be completed in December 2020 aims to establish the prime dose and tolerability of an HAP, tirapazamine, combined with embolization in HCC (clinicaltrials.gov Identifier: NCT02174549).

In addition to inhibiting hypoxia-induced signaling, improving HCC oxygenation could be an interesting approach to overcome sorafenib resistance. Indeed, some studies have analyzed the potential usefulness of a synthetic tetrameric hemoglobin, YQ23, which can target hypoxia to improve HCC therapy by its facilitation of oxygen delivery^51–53^.

Several drugs have been proposed as second-line treatment for advanced HCC after the failure of sorafenib therapy, and some of them are under evaluation in clinical trials^3,5^. Presently, only the tyrosine kinase inhibitor regorafenib (Stivarga^®^; Bayer HealthCare Pharmaceuticals Inc.; Leverkusen, Germany)^54^ and the human immunoglobulin G4 monoclonal antibody nivolumab (Opdivo^®^; Bristol-Myers Squibb Co.; NY, USA)^55^ have been approved by the FDA for HCC previously treated with sorafenib.

Further investigations focused on the elucidation of mechanisms involved in sorafenib resistance would allow for better understanding and help to propose more effective strategies to increase the efficacy of treatment.
